# Understanding the Rice Fungal Pathogen *Tilletia horrida* from Multiple Perspectives

**DOI:** 10.1186/s12284-022-00612-1

**Published:** 2022-12-16

**Authors:** Aijun Wang, Xinyue Shu, Deze Xu, Yuqi Jiang, Juan Liang, Xiaoqun Yi, Jianqing Zhu, Feng Yang, Chunhai Jiao, Aiping Zheng, Desuo Yin, Ping Li

**Affiliations:** 1 College of Agronomy, Sichuan Agricultural University, Chengdu, China; 2 Rice Research Institute, Sichuan Agricultural University, Chengdu, China; 3 Food Crop Research Institute, Hubei Academy of Agriculture Sciences, Wuhan, China

## Abstract

Rice kernel smut (RKS), caused by the fungus *Tilletia horrida*, has become a major disease in rice-growing areas worldwide, especially since the widespread cultivation of high-yielding hybrid rice varieties. The disease causes a significant yield loss during the production of rice male sterile lines by producing masses of dark powdery teliospores. This review mainly summarizes the pathogenic differentiation, disease cycle, and infection process of the *T. horrida*, as well as the decoding of the *T. horrida* genome, functional genomics, and effector identification. We highlight the identification and characterization of virulence-related pathways and effectors of *T. horrida*, which could foster a better understanding of the rice–*T. horrida* interaction and help to elucidate its pathogenicity molecular mechanisms. The multiple effective disease control methods for RKS are also discussed, included chemical fungicides, the mining of resistant rice germplasms/genes, and the monitoring and early warning signs of this disease in field settings.

## Background

*Tilletia horrida* Takahashi is a pathogenic basidiomycete fungus that causes rice kernel smut (RKS), a devastating grain disease in the production of rice male sterile lines in most hybrid rice-growing regions of the world. First reported in Japan in 1896 (Takahashi 1896), by the onset of the twentieth century RKS had already been found in India, Java, Siam, and China, and categorized then as a minor disease with sporadic occurrence in these rice-growing areas (Gade 2000; Giri et al. 2000; Akhtar and Sarwar 1987; Ayado et al. 1993; Iguchi et al. 1987; Chahal 2001; Biswas 2003; Carris et al. 2006). However, in order to ensure the higher seed production of rice male sterile lines, their exserted stigma has become increasingly common, resulting a greater incidence and impact of RKS (Webster and Gunnell 1992). RKS is now recognized as among the most crucial diseases globally affecting the majority of production areas cultivating hybrid rice varieties (Uppala et al. 2017; Chen et al. 2016). In China, the hybrid rice- growing area covers ca. 1.6 million acres, where the annual prevalence of RKS is 40% to 60% of these hybrid rice fields, with the disease causing 5% to 20% yield loss (Chen et al. 2016; Wang et al. 2015). In Pakistan, the disease incidence was reportedly as high as 87% in hybrid rice fields (Akhtar and Sarwar 1987). Currently, RKS poses a mounting threat to hybrid seed production across Asia, Oceania, Europe, America, and Africa (Brooks et al. 2009; Mohamed et al. 2014; Sharma 1999; Ribeiro et al. 1973; Gade 2000; Giri et al. 2000; Sharif et al. 2014; Ayado et al. 1993; Iguchi et al. 1987). In addition, there are several kinds of viewpoints about the classify of RKS during the past few decades, once lots of scholar regard RKS as *Neovossia horrida* (Huang et al. 2003, 1995); however, with the completion of genome sequencing together with the systemic identified of pathogenicity characters (Wang et al. 2015, 2018a, 2018b), RKS has been belonged to *T. horrida*.

**Fig. 1:**
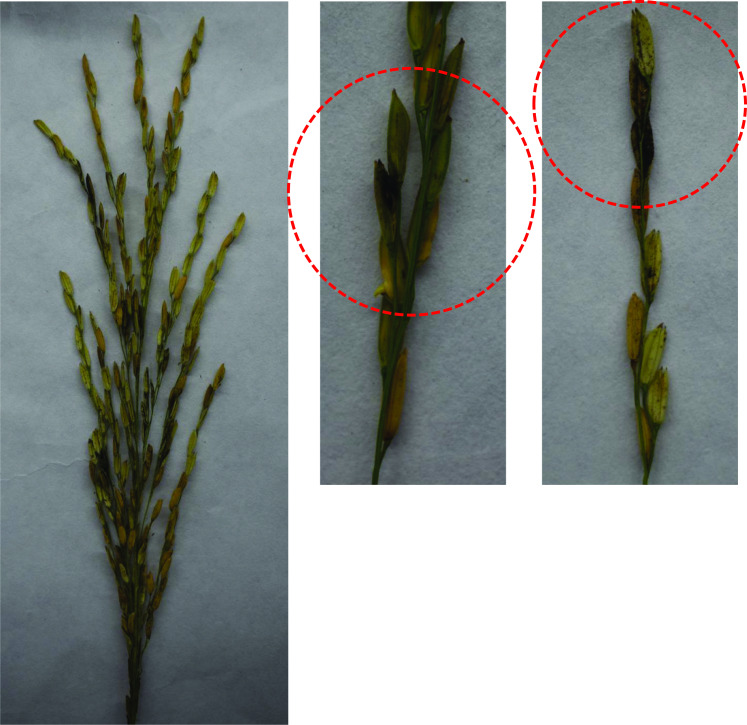
The symptoms of rice kernel smut disease

*T. horrida* infects rice flowers and colonizes their inner organs with mycelia, which eventually produce masses of dark powdery teliospores (Fig. 1). Teliospores are the main form of *T. horrida* during the overwintering period, and they can survive for more than 1 year in the soil, and at least 3 years on the surface of host seeds (Webster and Gunnell 1992). The early infective stage of *T. horrida* is asymptomatic (Zhu et al. 1998), and grain filling after pollination is blocked by the masses of dark powdery spores in the grains, thus causing a considerable reduction in the rice grain yield (Liu 2008).

Field control of RKS relies heavily on chemical fungicides and cultivation practices, and the breeding of rice male sterile lines resistant to this disease remains unsuccessful due to the paucity of robust resistance resources in the currently available rice germplasms. Moreover, environmental factors can affect the accuracy of RKS resistance phenotyping, and the ability of the pathogen to survive form one crop season to the next by forming teliospores makes it harder to control the fungal pathogen (Webster and Gunnell 1992; Teng et al. 2002; Dai et al. 2011). In light of this, here we also reviewed the control strategies, namely chemical and biological applications as well as the breeding for resistance traits in rice varieties.

In this current review, we summarize the pathogenic differentiation, disease cycle, infection process, functional genomics, and effector identification of the *T. horrida*. We also provide a thorough and critical discussion on rice defense responses to *T. horrida* infection and effective disease control strategies.

## Overview of Pathogens and Diseases

### Variability in Pathogenicity

Many plant pathogens have produced multiple physiological races whose virulence clearly differs during long the coevolution of pathogen–host interactions (Choi et al. 2013; Hamer et al. 1989; Kato et al. 2000). For example, *Magnaporthe grisea* that causes rice blast has dozens of physiological races. However, studies of the physiological races and pathogenic differentiation of *T. horrida* are quite scarce. Shao et al. (1997) inoculated two rice male sterile lines (Zhenshan 97A and V20A), each with four *T. horrida* strains separately, finding that the respective pathogenicity of these four strains was similar. Over the next 20 or so years, the number of rice male sterile lines bred has increased substantially, this providing stronger host conditions for both the pathogenic and population differentiation of *T. horrida*. Recently, Wang et al. (2018a) inoculated a susceptible rice male sterile lines (9311A) with seven *T. horrida* strains respectively isolated from different areas in China; their results showed these strains featured significantly pathogenic differentiation. Recently, the genetic diversity of the 63 T*. horrida* strains that isolated from different rice-growing areas in USA were investigated using multi-locus sequence analysis, which revealed the existence of five different groups of the *T. horrida* populations (Khanal et al. 2022). Collectively, these findings indicate that the *T. horrida* presently harbors significantly pathogenic and population differentiation.

### Disease Cycles

The smuts fungi are multicellular organisms, whose teliospores are widespread in soil and the seeds of host plants. To date, 80 smut genera (4200 species) with distinct morphological characters have been reported and all these infect higher plants, including many economically important crops, such as *Zea mays*, *Hordeum vulgare*, *Triticum aestivum*, *Oryza sativa*, *Saccharum officinarum*, and *Zizania latifolia* (Rogerson 1988; Stirnberg and Djamei 2016; Grewal et al. 2004; Imbaby and Eldaoudi 2002; Nasiru and Ifenkwe 2004). Those smut fungi parasitic on key crops have multiple phylogenetically separate lines, and include *Ustilago*, *Sporisorium*, and *Tilletia* spp. (Roux et al. 1998).

**Fig. 2:**
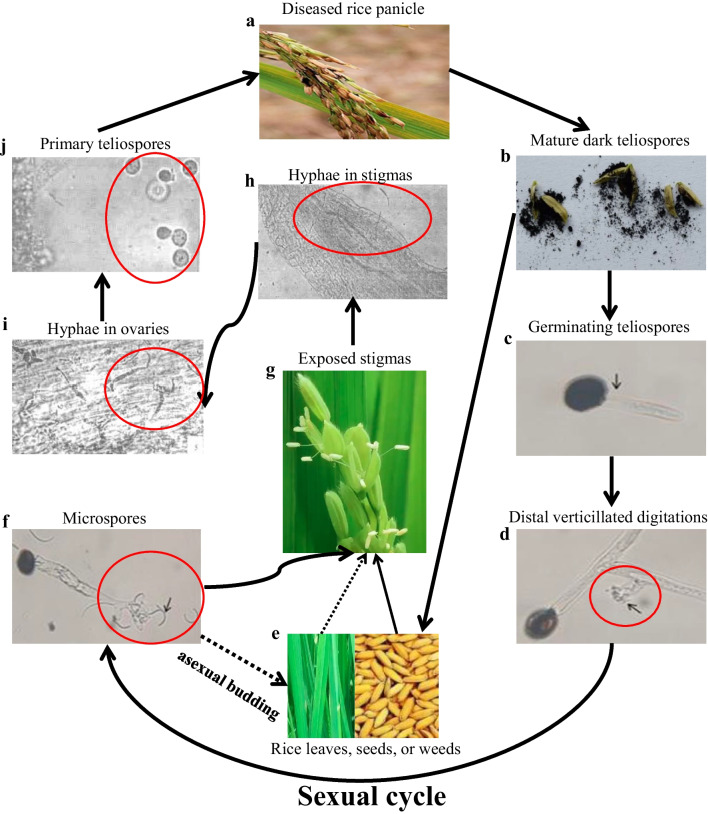
The disease cycle of rice kernel smut. **a** *Tilletia horrida* produces dark, powdery teliospores in the rice panicles. **b** Mature dark powdery teliospores. **c** Germinating teliospores. **d** Distal verticillated digitations. **e** Leaves or seeds of rice and paddy field weeds may exhibit epiphytic budding by *T. horrida*, which may contribute to the initial infection of rice flowers. **f** The secondary microspores of *T. horrida.* **g** The exserted stigma of rice male sterile lines. **h** Rice stigma infected by *T. horrida*. After removing to ovaries (**i**). **j** Primary teliospores were produced in ovaries. Shown are the paths for the sexual (solid lines) and asexual (dashed line) growth in the disease cycles. Figure adapted from Wang et al. (2018a) and Tao et al. (1998)

*T. horrida* of the *Tilletia* genus belongs to the basidiomycota Tilletiaceae family (Wang et al. 2015). As a biotrophic fungus, *T. horrida* can grow on artificial media and relies on a living host to reproduce, both sexually and asexually (Wang et al. 2018a). Under natural conditions, *T. horrida* is known to infect *Oryza sativa*. Through artificial inoculation tests of 32 species of grasses with *T. horrida*, it was found that *T. horrida* could infect *Oryza sativa* as well as *Aegilops sharonensis* (Royer and Rytter 1988). Furthermore, Singh et al. (1999) found that the secondary sporidia of *T. horrida* were capable of germinating on multiple non-host plants, such as *Cyperus rotundus*, *Echinochloa crusgalli*, *Zea mexicana*, *Sorghum vulgare*, and *Z. mays*. We provide an overview of the current understanding of asexual and sexual cycles of *T. horrida* (Fig. 2). After successfully infecting rice flowers, *T. horrida* produces many mycelia on the stigma and then infects other floral organs to form dark, powdery teliospores (Templeton 1961; Tao et al. 1998). These teliospores are round to elliptical in shape and possess colorless warty on their surface (Wang et al. 2018a, b, c, d, e). The diameters of teliospores are ca. 25–30 × 23–30 μm (Wang et al. 2018a). Thick-walled teliospores may sticks to ripe host seeds or to soil, and are disseminated by airflow and wind-blown rain in fields, where they survive over the winter. Abundant teliospores will germinate after overwintering under suitable temperature and humidity conditions, producing a promycelium that displays distal verticillated digitations (Wang et al. 2018a). Microspores capable of infecting rice flowers at the late booting stage grow in these verticillated digitations and the shape of these microspores is linear or curved (Wang et al. 2018a). Therefore, these secondary microspores serve as primary infection agents in the disease cycle, and they may exhibit epiphytic budding growth on the surface of not only host but also weed plants from the vegetative stage to flowering stage (Huang et al. 2003). Unfortunately, it is difficult to detect the germination process of overwintered teliospores in actual fields conditions. The function of overwintering teliospores in the disease cycle of *T. horrida* needs further in-depth investigation.

For *T. horrida*, like most basidiomycete fungi, the asexual reproduction stage in their life cycles is unimportant or absent entirely, instead reproducing sexually under field conditions. Early work indicated that meiosis of *T. horrida* occurs in the stage of basidium formation (Singh and Pavgi 1972). Additionally, *T. horrida* is heterothallic, as clearly proven by inoculation experiments using single and paired monospore lines of *T. horrida*, in that single basidiospore lines of *T. horrida* are avirulent (Singh 1998). After teliospores’ germination, the mononucleosis promycelium will form secondary hyphae through hyphal or basidium mating. Mitotic divisions of single haploid nuclei and subsequent formation of septa in the basidiospores after the formation of a basal septum delineating the basidiospore, results in two- to four-celled basidiospores (Carris et al. 2006). Each haploid cell of the basidiospore can produce hyphae to directly infect rice flowers.

### Infection Processes

Flower-infecting pathogens have evolved multiple strategies to infect their host plants. The pathogen responsible for rice false smut disease, *Ustilaginoidea virens*, specifically attacks the stamen filaments between the lodicules and ovaries; this results in significantly reduced growth of stamen filaments which precluded the formation of mature pollen, leaving the process of fertilization suppressed (Sun et al. 2020). Accordingly, *U. virens* infection induces the expression of many grains filling–related genes, such as grain starch biosynthetic genes and seed storage protein-encoding genes through imitate the process of ovules fertilization (Chao et al. 2014; Chen et al. 2010; Fan et al. 2015). This further activates the grain filling signaling pathway of rice, providing nutrients for the pathogen’s population growth. The study by Song et al. (2016) also showed that *U. virens* could hijacks the rice nutrients supply through blocking and imitating the fertilization of rice ovary. *Botrytis cinerea* infects alfalfa pollen grains mostly via pollen germ pores and goes on to obtains nutrients from the pollen exudates (Huang et al. 1999). Other phytopathogens which attack other parts of host flowers, such as their sepals, petals, and nectaries, have been summarized by Ngugi and Scherm (2006).

The infection processes of *T. horrida* was discerned through cytological observations of inoculation (Tao et al. 1998; Wang and Ouyang 1989; Zhu et al. 1998). Wang and Ouyang (1989) revealed that *T. horrida* hyphae spread over the ovaries of flowers through the stigmas and can reach nucellar tissue, and ultimately forming dark, powdery teliospores in aleurone cells or in the intercellular space between them. Tao and colleagues (1998) inoculated four rice male sterile lines (Zhenshan 97A, D90A, G46A, and K17A) with *T. horrida*, finding that the hyphae directly infected stigmas and further extended into nucellar tissue. Their research also demonstrated that *T. horrida* hyphae occur in ovaries at 8 h post inoculation (hpi), that primary teliospores form in the seed coat and intercellular space of aleurone cells at 7 days post inoculation (dpi), with teliospores maturating at 9 dpi. Interestingly, the process by which *T. horrida* infected the four rice male sterile lines was similar, not requiring pollination for infection (Tao et al. 1998). However, unlike *U. virens,* the teliospores formed only in ovaries that endosperm could normally fertilization (Zhu et al. 1998).

In rice, the floral organs’ tissue is not destroyed at the initial stage of *T. horrida* infection and lack obvious disease symptoms; the characteristics dark, powdery teliospores only appear at yellow maturity stage of rice (Zhu et al. 1998). This poses formidable challenge to early disease monitoring and further timely intervention of disease-controlling methods. Zhu et al. (1998) reported that *T. horrida* hyphae do not invade the cells and embryo sacs, such that the stigma cells and ovaries undergo no obvious visible changes and retain the normal ability of plasmolysis at 12 hpi. Collectively, these findings indicate that the infection strategy of *T. horrida* is unique and distinctive from other flower-infecting fungi.

### Pathogenic Mechanism of *T. horrida*

#### *T. horrida* Genome

Draft genome sequences of the *T. horrida* strain QB-1 were completed in 2015, by using Illumina Solexa GAII sequencing technology, resulting in an assembled genome size ca. 20 Mb in size, encoding 9038 predicted proteins (Wang et al. 2015). Later, the high-quality genome sequences of *T. horrida* strain JY-521 were obtained using the PacBio RS II sequencing strategy combined with three single-molecule real-time sequencing (Wang et al. 2018b). The assembled genome size of JY-521 is ca. 23.2 Mb and encodes 7729 predicted proteins, of which 6973 were supported by the RNA-seq data (Wang et al. 2018b).

Genome annotation and comparative genomics provide much crucial information pertinent to the pathogenic mechanisms of *T. horrida*. As a biotrophic pathogens, *T. horrida* has a genome with fewer carbohydrate-active enzymes (CAZymes) than some hemi-biotrophic and necrotrophic fungi (Wang et al. 2018b). Among these CAZymes are glycoside hydrolases, which break down cellulose and xylan in the plant cell wall, and pectin lyases that degrade pectin. The reduced polysaccharide degradation machinery indicates a diminished cell wall–degrading ability. This finding explains why *T. horrida* infection does not destroy the cells of stigmas and embryo sacs at early stage (Zhu et al. 1998).

In fact, biotrophic pathogens usually minimize CAZymes in their arsenal, to avoid the destruction of plant cell wall. The lysis of cell walls is often recognized by the host plant as a danger-associated molecular patterns, so ensuring an intact cell wall could avoid triggering plant immunity (Kämpe et al. 2006; Kemen et al. 2011; Zhang et al. 2014). This strategy is in accordance with the biotrophic lifestyle of *T. horrida* (Wang et al. 2018b). Although the *T. horrida* genome contains fewer genes related to cell wall degradation, it carries 1697 genes involved in pathogen-host interactions (PHI), which account for 21.96% of the total protein-coding genes (Wang et al. 2018b). Furthermore, *T. horrida* contains 4410 genes that have sequence similarity with those of four other smut fungi. Nevertheless, 2472 predicted genes are unique to *T. horrida* (Wang et al. 2018b). These findings could explain the differential hosts of smut fungi or diseases induced by these fungi, including *T. horrida*, *Ustilago hordei*, *U. maydis*, *Sporisorium reilianum*, and *S. scitamineum*. Importantly, the assembled genome of *T. horrida* and its annotation is especially valuable for the functional identification of pathogenicity genes and virulence effectors.

### Pathogenicity Pathway and Genes in *T. horrida*

Despite the genome of *T. horrida* having been sequenced and annotated, our knowledge of the molecular mechanisms underlying *T. horrida* virulence and pathogenicity is very limited. Functional genomics can contribute to the clarifying of pathogenic pathways and genes in *T. horrida*. Transcriptome analysis of the *T. horrida* virulent strain JY-521 at different times since its inoculation (8, 12, 24, 48, and 72 hpi) of susceptible rice accessions revealed 500 differentially expressed genes (DEGs) of which most were induced at 8 hpi (Wang et al. 2020a). This shows the primary host colonization by *T. horrida* occurred at the early stage of infection. Furthermore, KEGG (Kyoto Encyclopedia of Genes and Genomes) pathway analysis of these DEGs indicated that autophagy processes and lipid degradation were pivotal pathways involved in *T. horrida* pathogenicity (Wang et al. 2020a).

For successful colonization of plants, fungal pathogens generally secrete multiple kinds of CAZymes to break down the physical barrier of the host immune system (Cantarel et al. 2009). Thus, despite having fewer CAZymes in its genome, evidently these are critical for enhancing *T. horrida*’s virulence. Transcriptome analyses detected a carbohydrate esterases (CEs) family protein (*smut_2980*) involved in chitin deacetylase that was up-regulated at 12 hpi (Wang et al. 2020a). As previously reported, chitin deacetylase can prevent pathogenic fungi from being recognized by the plant immune system during the infection process (Gong et al. 2020; Sanchez-Vallet et al. 2020; Shimizu et al. 2010). Two glycosyltransferases (GTs) family proteins, *smut_1230* and *smut_1222* were also up-regulated at 8 hpi and may also play an important role during early infection of hosts. Furthermore, 64 genes related to PHI in *T. horrida*, such as *smut_1409*, *smut_3510*, *smut_6708*, and *smut_2974* were greatly induced during infection, suggesting these genes are likely involved in the infection of rice by *T. horrida* (Wang et al. 2020a). The conserved mitogen-activated protein kinases (MAPKs) have been widely identified as being pathogenicity factors in multiple phytopathogenic fungi (Dean et al. 2005; Soanes et al. 2007; Jiang et al. 2018). For example, *Ubc3*, a key gene involved in the MAPKs pathway of *Ustilago maydis*, is related to its hyphal growth and regulates its pathogenicity by interacting with Prf1 (Mayorga and Gold 2010). *Smut_0057* is the homeotic gene of *Ubc3* and may be associated with the pathogenicity of *T. horrida*. G protein-coupled receptors (GPCRs) and G-proteins are important proteins that involved in the MAPKs pathway (Dean et al. 2005; Soanes et al. 2007). Two nonsynonymous SNPs were found in the GPCR proteins *smut_1863* and *smut_4953* of the three weakly pathogenic strains vis-à-vis a strongly pathogenic strain, with *smut_4953* found up-regulated by *T. horrida* inoculation (Wang et al. 2020a). In addition, several putative secondary metabolites-related genes are up-regulated during host infection by *T. horrida* such as *smut_2974* that is associated with sterigmatocystin biosynthesis (Wang et al. 2020a). Hence, we speculated that sterigmatocystin is necessary for the pathogenicity of *T. horrida*. Besides, the cytochrome P450s-encoding genes associated with secondary metabolites possess many nonsynonymous SNPs when weakly versus strongly pathogenic strains are compared, implying that these genes contribute to *T. horrida* pathogenicity (Wang et al. 2018b; 2020a). These findings emphasize the essentiality of secondary metabolites for conferring pathogenicity to *T. horrida.*

### Secreted Proteins and Effectors

Effectors are crucial pathogenicity factors that suppress plant immunity and promote the successful infection of pathogens. Through comparative genomics and secretome analyses, 597 secreted proteins were predicted for the *T. horrida* genome (Wang et al. 2018b). Of the 597, 367 are small (< 400aa) cysteine-rich (SCR) secreted proteins; among the latter, the up-regulation of 131 SCRs was induced by *T. horrida* infection and these are considered candidate effectors (Wang et al. 2018b). The highly conserved effector gsr1 in *T. horrida,* which contains four conserved RNase active sites, is known to trigger cell death and an immune response in *Nicotiana benthamiana,* the former requiring the RNase active site (Wang et al. 2019). The effector uan2, which is unique to *T. horrida—*its homologous protein has not been found in other fungi*—*also triggers non-host cell death and an immune response when transiently expressed in *N. benthamiana*, and the predicted signal peptide of uan2 is essential for its cell death-inducing ability (Wang et al. 2019). Both effectors undergo up-regulation when *T. horrida* infects a host plant (Wang et al. 2019). Thus, according to transcriptome data, 26 putative effectors that presented the same expression pattern as gsr1 and uan2 were subsequently detected (Wang et al. 2019). The LysM effectors are widely distributed in plant pathogens and probably figure prominently in pathogenesis, with some identified in many fungi (Dölfors et al. 2019; Bolton et al. 2008; de Jonge et al. 2010; Gruber et al. 2011). In the *T. horrida* genome, seven putative LysM effectors (*smut_1650*, *smut_2305*, *smut_4126*, *smut_4802*, *smut_5680*, *smut_7273*, and *smut_7501*) have been annotated (Wang et al. 2018b). Recently, the putative LysM effectors *smut_1650* was shown to induce cell death in *N. benthamiana* (Shu et al. 2021). Moreover, some fungal glycoside hydrolases and chitinases were recently confirmed to function as effectors (Zheng et al. 2013; Han et al. 2019; Yang et al. 2019). For example, MpChi in *Moniliophthora perniciosa* encodes a secreted enzymatically-inactive chitinase consisting of 438 aa residues, to sequester chitin-triggered immunity. In *T. horrida*, a glycoside hydrolase family effector containing 268 aa residues with four cysteines has been identified to trigger non-host cell death and an immune response (Shu and Wang unpublished data). Interestingly, like uan2, multiple predict effectors that induce non-host cell death and an immune response unique to *T. horrida* have been predicted to exist (Jiang and Wang unpublished data). To sum up, the effectors in *T. horrida* fulfill important roles in its pathogenicity. The knock-out of effector proteins-encoded gene in *U. virens* also confirmed its functioning in conferring virulence and pathogenicity to this biotrophic pathogens (Sun et al. 2020). Next, it is imperative we identify the pathogenicity functions of effectors in *T. horrida* by using gene-knockout techniques, and more importantly, to identify the host targets of these *T. horrida* effectors, which could be utilized as elegant probes for the identification of potentially resistant proteins in plants.

## Management and Control Measures

As the incidence of RKS increases, measures for the effective control of this disease are urgently needed. In this respect, researchers have tried many approaches (Biswas 2001; Wang et al. 2018a, b, c, d, e; Yang et al. 2022; Jiang et al. 2021; Nataraja 2002; Brinck and Gärdenfors 2007), including the mining of resistance traits in rice cultivars or genes, chemical fungicides control, different cultivation practices, and better monitoring protocols and early warning systems. Yet research into breeding resistance in rice to RKS is proceeding slowly.

### Searching for Resistant Rice Cultivars or Resistance Genes

Detecting the RKS-resistant rice cultivars or genes and then breeding disease-resistant lines are considered the most efficient, economical, and environmentally friendly methods for disease control. But it is difficult to assess RKS resistance in field settings due to immature inoculation methods and the instability of environmental conditions. Natural infection in the field is one of the most commonly used methods to evaluate the disease resistance of a crop (Wang et al. 2018a, b, c, d, e; Deng et al. 1997). To evaluate RKS, the rice plants are grown in a field under standard field management conditions without any fungicide application. Then, at crop maturity, the incidence of RKS disease is determined by counting the number of single-ear affected grains and deriving an infected seed rate, or by using evaluation criteria as described by Deng et al. (1997). It should be noted that such natural infection experiments should be performed across years and sites because the RKS incidence varies under different environmental conditions. However, the identification of RKS-resistant rice plants through artificial inoculation under controlled conditions is more efficient and reproducible than pursuing it under natural infection (Cartwright et al. 1996). The methods of artificial inoculation mainly include spore suspension spraying and single floret injection. Concerning the former, inocula were produced with ∼5–7 days, through mycelia of *T. horrida* grown on potato sucrose fluid medium, and spore concentration is adjusted to 1 × 10^6^ conidia/mL with sterilized potato sucrose fluid medium. At the blooming stage of a rice plant, the spore suspension is sprayed onto the stigma of a floret with a pocket-sized sprayer, but the glume must be cut open and the stigma was exserted. For floret injection, the spore suspension is injected into a rice floret with a syringe at the late booting stage. After inoculation, rice male sterile lines need to be pollinated and are maintained at 28 °C and at least 95% relative humidity. Several rice male sterile lines featuring resistance to RKS in the field have been detected and these may be used in mining resistance genes and disease-resistance breeding through artificial inoculation experiments (Wang et al. 2018a, b, c, d, e; Zong et al. 2004; Akhtar and Sarwar 1988). The RKS resistance of 16 rice cultivars was determined at the anthesis stage using an artificial inoculation method: the disease incidence ranged from 6.25% to 97.25% at Sheikhupura and from 5.25% to 95.25% at Gujranwala, with the C-622 cultivar presenting the highest resistant level (Akhtar and Sarwar 1988). Earlier, the rice line IR-579 was also found to harbor resistance to *T. horrida* (Singh and Pavgi 1970). Yu and colleagues (2004) examined the RKS resistance of 10 photo-thermo-sensitive genic male sterile rice lines, found that their resistance levels differed markedly, with line S25 not infected by kernel smut disease. Recently, Wang and colleagues (2018c) evaluated the RKS resistance of 78 rice male sterile lines in the field over 3 years; their results also indicated that the resistance levels of different rice lines varied significantly, among which four lines that present more than medium resistance 4766A, Jiangcheng3A, Jufeng2A, and TianfengA were obtained. However, genes or quantitative trait loci (QTLs) involved in RKS resistance are rarely discussed (Dai et al. 2011; Liu 2008). Using the F_2_ population from a cross between the resistant line Jiangcheng 3B and the highly susceptible cultivar 9311B, resistance QTLs were mapped to chromosome 6 and 11 by bulk segregate analysis, and this further identified a resistant gene located in chromosome 11 when combined with transcriptome and transgenesis techniques (Wang and Zheng unpublished data). Recently, five QTLs for RKS resistance were mapped using a recombinant inbred lines population constructed by the resistant cultivar W9593S and the susceptible cultivar Aipei64S, using a composite interval mapping method (Yang et al. 2022). Among these five QTLs, qRRKS-11 and qRRKS-8 respectively explained 13.64% and 12.07% of the phenotypic variation (Yang et al. 2022).

The disparate transcriptome responses between resistant and susceptible rice male sterile lines against *T. horrida* infection indicate that encoding calmodulin-like proteins genes *OsCML7* and *OsCML14*, involved in reactive oxygen species burst genes *Os09g0467200*, *Os01g0369700*, and *Os01g0949800*, the NADPH oxidase encoded gene *Osrboh9*, and the salicylic acid signaling pathway-related gene *OsNPR1* may all contribute to RKS resistance (Wang et al. 2020b; 2018d). Interestingly, two flowering time regulation genes in rice, *OsCTR2* and *OsRR1*, were exclusively up-regulated in the resistance line after *T. horrida* inoculation, and the overexpression these two genes resulted in a late-flowering phenotype (Wang et al. 2013; Cho et al. 2016). And flowering time regulation genes *OsABF1*, which RNA interference lines present late-flowering phenotype (Zhang et al. 2016a, b), was down-regulated in resistance line, but not induced expression in susceptible line. Field observations suggest that rice cultivars with a short-duration of flowering often have a greater kernel smut disease incidence than do long-duration flowering cultivars. Muthusamy and Ahmed (1977) detected the responses of 10 early-maturing and 9 later-maturing rice varieties to infection with *T. horrida*, finding that those with a shorter growing season were more likely to get infected than those with a longer growing season. Therefore, the delaying of flowering or start of the growing season should be considered in the control of RKS in rice production.

### Chemical Fungicides Control

In fact, RKS disease control largely relies on fungicide applications during the typical production of hybrid rice (Tang et al. 2010; Feng and Lu 2009; Grewal et al. 1996; Hakro et al. 1988). Among many of tested fungicides, trifloxystrobin tebuconazole WG, benazoxystrobin SC, acetobacter fluconazole, diniconazole carbendazim WP, Keheijing, and Kanghei 95 are all highly effective against *T. horrida* infection and have been utilized in controlling kernel smut disease (Ding et al. 2020; Ye et al. 2005; Zhang et al. 1998). The ensuing control effect is often higher for mixed application of multiple fungicides than a single one. For example, Kanghei 95, a mixture of Jinggangmeisu, carbendazim, triadimefon, KH_2_PO_3_, and sodium borate, exerted a preventive effect against RKS of more than 90%, which exceeded that obtained by the individual application of Jinggangmeisu and carbendazim (Ye et al. 2005). In particular, the timing of fungicide application is pivotal for high efficacy in the control of RKS. The best practices for fungicide spraying in RKS management is to apply once from the end of boot stage to the initial heading stage, and once again at the early flowering stage, and one more time at the peak flowering stage, respectively (Honkura and Osada 1993; Yang et al. 2001; Shu et al. 1993). Additionally, using a biotic pesticide is an environmentally friendly methods to control plant diseases. It is well-known that many *Bacillus subtilis* and *Trichoderma* strains are used to successfully manage a suite of plant diseases (Andargie et al. 2017; El-Naggar et al. 2015). For example, several *Trichoderma* spp*.*, such as *T. viride*, *T. harzianum*, and *T. koningii* have high rice false smut control efficiencies ranging from 46.8% to 100% (Andargie et al. 2017; El-Naggar et al. 2015; Liang et al. 2014). For *T. horrida*, El-Kazzaz et al. (2015) found that a *Bacillus pumilus* strain markedly presented promising characteristics for controlling the RKS. However, biocontrol microorganisms that control RKS have not been described; hence, it is necessary to strengthen this aspect in future studies.

### Cultivation Practices

Excessive applications of nitrogen fertilizer and a high humidity environment will promote the incidence of RKS (Slaton et al. 2004). Thus, curbing nitrogen fertilization and modified planting density, as well as establishing a field microenvironment that is detrimental to the pathogen’s growth and development, could be effective measures to control RKS in rice production. Moreover, the teliospores of *T. horrida* can overwinter epiphytically on surviving rice plants, and act as the primary source of infection the next year. Accordingly, eliminating plants’ survival in field and irrigation canals could reduce the primary infection reservoir of RKS. The paddy (rice)-upland rotation (with a drought crop, such as maize), along with furrow irrigation, rational fertilization, appropriate early sowing, and cultivating strong cuttings are also effective at suppressing the occurrence rate of RKS (Slaton et al. 2007; Brooks et al. 2009; Kalboush et al. 2018).

### Monitoring and Early Warning

Because obvious disease symptoms are absent during the initial stage of *T. horrida* infection, implementing control measures when the symptoms appear is too late to avoid yield losses. Thus, monitoring protocols and early warning systems are both very important for managing kernel smut in situ. Chen et al. (2016) reported on specific internal transcribed spacer (ITS) primers, which can be used to detect the presence of *T. horrida* in the floret of rice at the early inoculation stage. This finding could be very useful for devising rational and effective control measures. In fact, integrated multiple management methods are recommended for those rice-growing areas incurring a high kernel smut incidence.

### Study of Tilletia Species

The genus *Tilletia* is a grass disease fungus infecting cereal crop either locally or systemically (Carris et al. 2006). The cereal-infecting *Tilletia* species that forms teliospores in the ovaries of their hosts are defined as bunt fungi (Bishnoi et al. 2020; Muhammad et al. 2013). Among these *Tilletia* species, there are five pathogens capable of infecting economically important crops; except *T. horrida*, the other four can also infect wheat plants. Although *T. caries* and *T. laevis* are responsible for common bunt of spring and winter wheat crops, the teliospore wall structure of these two fungi is not the same: *T. laevis* has a smooth surface and *T. caries* has a reticulated surface (Zhang et al. 2002). *T. controversa* is a quarantine-listed pest in many countries and causes dwarf bunt of autumn-planted wheat, yet it has never been found on spring-planted wheat (Xu et al. 2021). The symptoms caused by *T. controversa* in wheat are similar to *T. caries*, but these two smut pathogens differ in the composition of their teliospore structures (Zhang et al. 2002). The teliospores of *T. controversa* have a conspicuous hyaline gelatinous sheath whose thickness is 1.5–5.5 μm. The paramount smut fungus infecting wheat is Karnal bunt, caused by *T. indica*, whose teliospores also are covered by a conspicuous hyaline gelatinous sheath, and densely echinulate or finely cerebriform distributed on their surface (Pady et al. 1961; Zhang et al. 2002). Moreover, like *T. horrida*, these four fungi also survive on the seed surface of host seeds and in soil, and diseased seeds are the most important potent source of infection. However, these four pathogens infect wheat hosts at the seedling stage, in stark contrast to *T. horrida* (Zhang et al. 2002).

The high-quality genome sequences of four Tilletia species mentioned are now available. The assembled genome size of *T. controversa*, *T. caries*, *T. laevis*, and *T. indica* is ca. 49.87, 35.8, 28.78, and 37.46 Mb, these encoding 10459, 10043, 9799, and 9664 predicted proteins, respectively (Kumar et al. 2017; Gurjar et al. 2019; https://ncbi.nlm.nih.gov/). The genetic diversity of the 20 T*. indica* strains that isolated from different locations in Indian were also detected using seven multilocus sequence fragments, the results revealed that the population of *T. indica* was highly diverse (Gurjar et al. 2021). However, functional research of the virulence or effector proteins in these smut fungi very limited. For the control of these diseases, like that caused by *T. horrida*, much progress has been made by researchers (Kumar et al. 2016; Tan and Murray 2006; Gupta et al. 2019; Singh et al. 2020; Gurjar et al. 2022). For example, many wheat varieties, including Paroli, Gluten, NGB-9015, Maribos, PG3540, and Kranich, are described as being resistant to *T. caries*, and several major genes in them have been are identified as able to control this disease (Gupta et al. 2019). Synthetic hexaploid wheats, derived from *Triticum turgidum* × *T. tauschii*, is resistant to *T. indica* (Villareal et al. 2010). With the rapid development of high-throughput technologies, more genome-wide association studies (GWAS), bulk segregant analysis (BSA) and transcriptomic analyses, have been carried out to to detect the genes or locus that are resistant to these smut diseases in wheat (Singh et al. 2020; Gupta et al. 2019). Furthermore, a loop-mediated isothermal DNA amplification tool that can used be to quickly detect *T. caries*, *T. laevis*, *and T. controversa* in wheat grain was also reported on (Pieczul et al. 2018). This provides a crucial method to manage these smut diseases through their in situ monitoring in fields as part of an early warning system.

## Conclusions

RKS has emerged as globally important disease, causing serious yield losses to hybrid rice seed production in recent years. Developing disease-resistant varieties using resistant genes is often viewed as the most economic and effective strategy to control this worsening disease threat. However, no RKS resistance gene has yet been identified and there are only few reports of mapping of RKS resistance QTLs, leaving the major QTLs with high phenotypic variance unfound. Consequently, we lack reports addressing RKS resistance breeding that would utilize those QTLs. The principal reason for this phenomenon is less innate resistance in rice germplasms and mapping populations currently available for RKS study. But now, with the rapid development of sequencing technology, it is easier to explore natural variation that associated with RKS resistance in hidden defense-related QTLs or genes among rice male sterile lines. Therefore, upcoming studies should focus on detecting effects of QTLs or genes that are involved in the RKS resistance trait, and further pyramiding several favorable consistent QTL alleles into a single cultivar to enhance the overall RKS resistance of rice male sterile lines is crucial.

Researching the function of effectors or virulence factor genes from *T. horrida’*s genome sequence has significantly contributed towards a better understanding of its pathogenesis and for subsequently developing sound management strategies. Although the candidate effectors of *T. horrida* have been predicted based on its available genome sequence (Wang et al. 2018a, b, c, d, e), their transgene study has not been performed to characterize its pathogenicity. The CRISPR-mediated genome editing technologies have enormous potential to validated the pathogenic functioning of effectors in fungi (Molla and Yang 2019; Pickar-Oliver and Gersbach 2019). To accomplish this target, a standardized genetic transformation system of *T. horrida* needs to be established.

Furthermore, to enhance the disease resistance of rice germplasms, impairing expression of pathogenicity-related genes in pathogen by using their small interference RNAs (siRNAs) sequences inserted into rice plants is a promising strategy. For example, the UvAspE, UvCom1, and UvPro1 are three virulence-related proteins of *U. virens*, for which the insertion of their siRNAs’ fragments into the rice cultivar Nipponbare significantly augmented its resistance to *U. virens* (Chen et al. 2021). The role of effectors siRNAs of *T. horrida* in RKS resistance are still poorly explored, however. Thus, analyzing the role of *T. horrida*’s effectors siRNAs in conferring host resistance would significantly advance our understanding of how to better manage this disease.

Detecting resistant proteins that recognize pathogen effectors using modern molecular biology experimental techniques, such as yeast-two-hybrid system, bimolecular fluorescence complementation, and co-immunoprecipitation (Wang et al. 2018a, b, c, d, e) will also help to control RKS and improve our understanding of *T. horrida*–rice interactions. Yet such study is hindered by the lack of virulence gene resources. Thus, we should enhance the study of antifungal proteins, which recognize pathogen effectors, as this could provide insight for clarifying the molecular mechanism underpinning the interaction of *T. horrida* and rice. Despite all this, any possible methods that reliably control RKS disease should not be ignored in the near future.

Like RKS, the diseases caused by *T. controversa*, *T. caries*, *T. laevis*, and *T. indica* are problematic, causing economical losses by extensively impairing wheat crop health and quality worldwide. For these *Tilletia* fungi, mechanism study of the molecular interaction between the pathogen and host are also limited. As mentioned above, the future studies should focus on distinguishing the effects of resistant QTLs or genes, the function of effectors based on the published genomic data of the involved *Tilletia* fungus, and further identify potential resistant proteins in host plants. More important is applying resistant genes of host and siRNAs of pathogen effectors to resistance breeding effectively, this being a necessary measure to settle the problem to food security posed by these diseases. In this way, not only may we gain the effective preventive and control of these smut diseases but also better understand the molecular mechanisms of their interactions between host and pathogen.

## Data Availability

Not applicable.
